# An integrated knowledge translation approach to address avoidable rehospitalisations and unplanned admissions for older people in South Australia: implementation and evaluation program plan

**DOI:** 10.1186/s43058-021-00141-w

**Published:** 2021-04-07

**Authors:** Gillian Harvey, Clarabelle T. Pham, Maria C. Inacio, Kate Laver, Elizabeth A. Lynch, Robert N. Jorissen, Jonathan Karnon, Alice Bourke, John Forward, John Maddison, Craig Whitehead, Jesmin Rupa, Carmel McNamara, Maria Crotty

**Affiliations:** 1 College of Nursing and Health Sciences, Flinders University, Adelaide, Australia; 2 Flinders Health and Medical Research Institute, Flinders University, Adelaide, Australia; 3 Registry of Senior Australians, South Australian Health and Medical Research Institute, Adelaide, Australia; 4 Division of Rehabilitation, Aged and Palliative Care, College of Medicine and Public Health, Flinders Medical Centre, Flinders University, Adelaide, Australia; 5 Department of Geriatric and Rehabilitation Medicine, Royal Adelaide Hospital, Adelaide, Australia; 6 Aged Care, Rehabilitation and Palliative Care Division, Northern Adelaide Local Health Network, Adelaide, Australia; 7 Medical Services, Northern Adelaide Local Health Network, Adelaide, Australia; 8 Adelaide Nursing School, University of Adelaide, Adelaide, Australia

**Keywords:** Hospitalisations, Unplanned rehospitalisations, Emergency department encounters, Older people, Implementation, Evaluation, Integrated knowledge translation, Quality improvement

## Abstract

**Background:**

Repeated admission to hospital can be stressful for older people and their families and puts additional pressure on the health care system. While there is some evidence about strategies to better integrate care, improve older patients’ experiences at transitions of care, and reduce preventable hospital readmissions, implementing these strategies at scale is challenging. This program of research comprises multiple, complementary research activities with an overall goal of improving the care for older people after discharge from hospital. The program leverages existing large datasets and an established collaborative network of clinicians, consumers, academics, and aged care providers.

**Methods:**

The program of research will take place in South Australia focusing on people aged 65 and over. Three inter-linked research activities will be the following: (1) analyse existing registry data to profile individuals at high risk of emergency department encounters and hospital admissions; (2) evaluate the cost-effectiveness of existing ‘out-of-hospital’ programs provided within the state; and (3) implement a state-wide quality improvement collaborative to tackle key interventions likely to improve older people’s care at points of transitions. The research is underpinned by an integrated approach to knowledge translation, actively engaging a broad range of stakeholders to optimise the relevance and sustainability of the changes that are introduced.

**Discussion:**

This project highlights the uniqueness and potential value of bringing together key stakeholders and using a multi-faceted approach (risk profiling; evaluation framework; implementation and evaluation) for improving health services. The program aims to develop a practical and scalable solution to a challenging health service problem for frail older people and service providers.

Contributions to the literature

- This study protocol introduces a state-wide, multi-sectoral approach to tackling a complex problem that affects older people at high risk of repeated hospital admission.
- The study leverages existing registry data to inform the design and evaluation of older person-centred improvements in care.
- The three inter-linked work packages (risk profiling; evaluation framework; implementation and evaluation) underpinned by an integrated approach to knowledge translation, with extensive stakeholder engagement, optimises the potential for sustainable improvement in health services.

## Background

Avoidable hospitalisations of older people remain a global challenge for health care systems [1–3]. Additionally, the number of unplanned visits to emergency departments (ED) has continued to grow even though many countries have achieved substantial health care improvements in recent decades [4–6]. Increased rate of hospitalisations is burdensome for health care systems and undesirable for patients, particularly for older people with multiple co morbidities [7].

Existing literature identifying the risk factors associated with hospitalisations indicates that there are no simple solutions. Studies have identified socio-demographic and environmental factors such as higher age, being male, level of educational attainment, marital status, geographical location, socio-economic deprivation, living alone without help, reduced social activity, and lack of community engagement as potential risk factors for hospitalisations and ED presentations [2, 8–16]. Additionally, a wide range of medical conditions and health care-related factors such as self-rated poor health status, frailty, frequent falls, co-morbidity, polypharmacy, overuse and underuse of medications, depression, anxiety, heart failure, cognitive impairment, higher number of primary care visits, and admission to nursing home have also been identified [5, 17–22]. However, few studies have examined the combination of individual, medication, system, and hospital-associated factors that together could be associated with frequent hospital admissions.

A previous history of hospitalisations is associated with a higher risk of avoidable rehospitalisations, particularly in older people aged 65 and above [11, 23]. An avoidable rehospitalisation occurs when a patient who has been discharged from hospital is admitted again within a certain time interval, where the readmission could potentially have been avoided through improved clinical management or appropriate discharge planning [23, 24]. Unplanned hospital readmissions are important to patients, families, clinicians, and policy makers since reducing their occurrence can improve care and reduce the costs of health care [8]. However, the evidence of policy interventions that effectively reduce unplanned hospital readmissions in older people is sparse.

The importance of collaboration between the spectrum of care providers such as hospital, aged care, local agencies, and primary care [25–27] is highlighted in the literature to support interventions aimed at reducing unplanned rehospitalisations. Moreover, patient education, home follow-up, home monitoring, adjustment of medication, and regular communication with clinical experts have been found to be effective in reducing unplanned rehospitalisations in older people [28]. A recent systematic review suggested that self-management, telephone follow-up, and medication reconciliation activities are most likely to be effective in reducing hospital readmission [29].

Similar to other international experiences reported in the literature, in Australia, older people with multiple long-term conditions have complex health and social care needs, which increases their risk of repeated hospitalisations. Australians aged 65 years and over represent 15% of the population yet account for 20% of ED presentations and 42% of same day hospitalisations; people aged 85 years and over account for 23% of all the presentations for people aged 65 years and over [30]. Many hospital admissions occur for ambulatory care sensitive conditions where hospital admission could have been prevented by interventions in primary care [31, 32].

People aged 65 and over represent 18% of South Australia’s (SA) population, which is higher than the national average [30]. Current approaches to addressing the problem of avoidable hospital admissions and readmissions in SA differ between various administrative structures within the state. For example, some health services for the older population are only available within certain local health networks (LHNs), and evaluation criteria between services differ. Therefore, it is unclear which services are most appropriate and effective for which patients and at what cost to individuals and the state health service.

The State Action on Avoidable Rehospitalisations and Unplanned Admissions across South Australia (STAAR-SA) program was designed to establish a state-wide approach, to better understand the problems and design, implement, and evaluate appropriate local solutions. The STAAR-SA program is applying a co-design approach, engaging with different stakeholder groups (e.g. consumers, front-line clinicians, managers) throughout the study to embed an integrated Knowledge Translation (iKT) approach [33, 34]. The benefit of the iKT approach is that the expertise and skills of both the researchers and knowledge users are explicitly recognised, and through collaboration, any discrepancies between the existing evidence and the needs of the health service can be identified and subsequently addressed [35, 36]. The program involves the stakeholders and researchers in three key research activities to: profile the 65+ population most at risk of re-hospitalisations and unplanned admissions in South Australia; evaluate current out-of-hospital programs on offer for older people; and translate evidence into practice to evaluate through a quality improvement collaborative (QIC). In this way, an iKT approach is intended to generate more rapid translation and enhanced impact.

## Methods

### Study aims

**Fig. 1:**
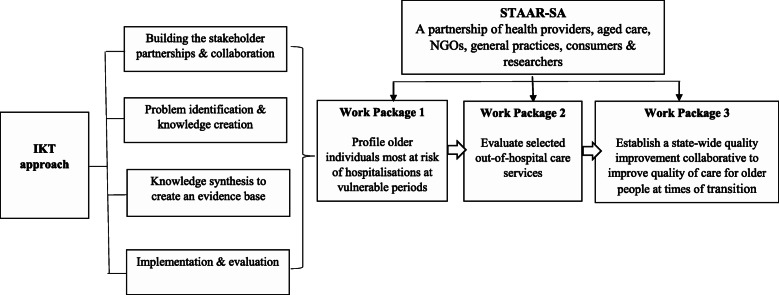
Project aims mapped against an integrated Knowledge Translation (iKT) approach

The overarching aim of the project will be addressed through three inter-connected research activities which are referred to as “Work Packages” (Fig. 1).

Work Package 1 (WP1) aims to construct a series of (six) predictive models for three different outcomes (unplanned hospitalisation, emergency department encounters and mortality) at two vulnerable time periods for older people, namely, time of an aged care eligibility assessment or entry into permanent residential aged care, and identify opportunities to reduce morbidity and health care utilisation. Each model will examine whether individual-related characteristics, medication-related characteristics, system-related characteristics, and hospital-related characteristics are predictive of the outcome of interest. Once the predictive models are completed, the aim will be to develop and validate risk profiling tools for hospitalisations and emergency department encounters that can be used for decision-making support, individualisation of care plans, and educational purposes for individuals at vulnerable periods in their aged care journey.

Work Package 2 (WP2) comprises the following objectives to evaluate selected existing out-of-hospital care services in South Australia: determine the outcomes (primary care utilisation, hospital admissions and ED presentations) from routinely collected 12 months data after commencing an out-of-hospital care program, compared with the same outcomes in (a) the same patient in the two years prior to commencing the out-of-hospital care program and (b) a control group (who did not commence the program) selected from the Registry of Senior Australians (ROSA) and develop an evaluation framework to provide a standardised approach for the monitoring and reporting and assessment of the costs and outcomes of out-of-hospital care services. WP2 will also include a qualitative component to explore the consumer/carer experiences of the selected out-of-hospital services to inform and provide context to the quantitative component.

Work Package 3 (WP3) will involve the establishment of a state-wide QIC to improve the quality of care for older people at times of transition. The QIC will use the findings from WP1 and WP2 to guide their improvement activities. The QIC model is based on evidence that assessing one’s own progress and benchmarking with other professionals can facilitate faster and wider implementation of quality improvement practices [37]. We will replicate in part the STAAR improvement collaborative from the USA, utilising the four-point change model: (1) improving assessment of post-hospital needs, (2) educating patient and family caregivers, (3) following-up post-hospital care (both medical and social services), and (4) communicating critical information of the patients transitioning to the next clinician or health care services [38].

### Context and setting

The project includes the three metropolitan and six regional LHNs, the two state-based primary health networks (PHN) that coordinate health services in local areas, three aged care providers, one consumer organisation and academics from the three largest SA universities and the South Australian Health and Medical Research Institute (SAHMRI). Prior co-designed research undertaken by project team members to explore the experiences and perspectives of older people, their families, carers, and service providers in relation to managing their condition, remaining well at home, and avoiding unnecessary hospitalisation, also provided important background information for the current study [39, 40].

### Participants

WP1 will utilise an existing data registry, the Registry of Senior Australians (ROSA), which includes longitudinal information on pathways and transitions in aged care linked with participant encounters with the health care sector and mortality. ROSA has a national historical (1997–2016, 2.9 million participants) and a prospective SA-based (2018–ongoing, 16,000 participants/year) cohort. WP1 will include any individuals who either completed an Aged Care Assessment Team (ACAT) assessment between January 2012 and May 2016 or entered permanent residential aged care between 2013 and 2016 (who also have timely ACAT data).

In WP2, out-of-hospital care services aimed at managing people with chronic conditions to reduce unplanned rehospitalisations in the metropolitan and regional LHNs in South Australia will be evaluated. This will be done through the collation of data on a retrospective cohort of patients who have received a service and comparing them with a control group (similar patient characteristics but have not received the service) within ROSA. WP2 will include South Australian individuals aged 65 years and over who have had an ACAT assessment to enable linkage to ROSA.

For WP3, health and aged care teams will be invited to participate in the QIC through a combination of purposive and snowball sampling. Teams may be working in acute, primary care or community settings and employed by government or non-government organisations.

### Measures

In each of the two cohorts of WP1 (people who have had an ACAT assessment, people admitted to permanent residential care), the study includes individual, medication, system, and hospital-related characteristics. Individual-related characteristics include age, gender, marital status, health conditions, veteran and concession card status, cognitive impairment, depression status, activities of daily living limitations, and behavioural and complex health care needs. Medication-related characteristics include polypharmacy, dispensing of medications known to be associated with hospitalisations, and any potentially inappropriate medications in older people. Facility/provider characteristics, geographical area, state, and year are included as system related characteristics. Finally, history of hospitalisations and emergency department encounters, history of primary care and specialist encounters, time between previous hospital discharge, and ACAT assessment will be examined as hospital and health care-related characteristics.

For WP2, potentially eligible out-of-hospital services will be nominated by LHNs, PHNs, SA Ambulance Service, and NGOs. To be eligible for the quantitative evaluation, services have to have commenced before December 2017, have an adequate sample size of at least 100 patients enrolled by December 2017, and have patient identifiers (full name, date of birth and Medicare number or hospital admission date) to enable linkage to the historical ROSA dataset. Data on the retrospective patient cohorts from included out-of-hospital care services will be analysed.

In WP3, we will convene a panel of experts (representing a variety of health and aged care providers, consumers, and academics). The expert panel members will be presented with the latest evidence about hospital transitions from both quantitative and qualitative studies and then tasked with identifying the main modifiable problems which contribute to avoidable hospital readmission in SA and suggest specific strategies to manage these. The panel will also be asked to identify outcomes or process indicators that can be used to monitor change over time. Once the main modifiable problem is identified, the researchers will advertise amongst the health and aged care services in SA to recruit teams to participate in the QIC. The QIC members will participate in three learning meetings over a period of approximately nine months. The researchers will then evaluate the success of the QIC by (1) measuring changes in adherence to selected process indicators within each service, (2) assessing levels of improvement knowledge in collaborative members—pre and post involvement, (3) conducting interviews and/or focus groups to ascertain attitudes towards the collaborative, and (4) measuring new linkages between key personnel using social network analysis.

### Outcomes

The anticipated outcomes from the Work Packages of this study are presented in Table 1.

**Table 1:** STAAR-SA potential project outcomes

Work packages	Outcome details
WP1 *Risk profile*	Risk profiling tools for hospitalisation. For each of the following outcomes, the time is measured from either date of completion of aged care eligibility assessment (cohort 1) or date of entry into permanent residential aged care (cohort 2). - Time to unplanned hospitalisation after entry into the relevant cohort - Time to ED encounter - Time to death
WP2 *Evaluation framework*	For each out-of-hospital care service: - Cost per unplanned hospitalisation - Cost per bed day saved (includes the costs for hospitalisation, emergency department presentations and primary care) - Consumer/carer experience - Mortality - The extent to which the program reaches the intended target group - Variations in implementation of the program between different population recipients
WP3 *Implementation and evaluation outcomes*	- Establishment of a network of people working in out-of-hospital care in SA - Formation of linkages across LHNs, primary care settings, and non-government aged care providers - Increased adherence to a core set of quality process indicators for transitions in care for older people - Improved quality of care transitions - Increased capability for quality improvement

### Data analysis

Data analysis will be both quantitative and qualitative in nature, based on the individual Work Package objectives.

The quantitative analysis of cohort and crude outcomes in WP1 will be described using means, standard deviation, medians, interquartile ranges (IQR), frequency, and proportions. For most predictive models for hospitalisation and ED encounters, a Fine-Gray model will be employed with death as a competing risk. For predictive models for mortality, Cox regression/Lasso will be employed [41]. The proportional hazards assumptions will be tested using Schoenfeld residuals. Prediction models will be assessed by application to training set data (January 2013–May 2016) using 10-fold cross-validation. Sub-distribution hazard ratios and 95% confidence intervals will be presented for examined factors found to be predictors of the outcomes of interest. The models’ calibration will be examined by comparing observed and predicted mean survival where the groups are defined over quantiles or deciles of the predictions at specific time periods. Discrimination will be examined using Harrell’s C-index [42].

In WP2, prospective data collection involving qualitative interviews with older people to understand their experience of the out-of-hospital services will be subject to thematic analysis [43]. Interrupted time series (ITS) analysis will also be performed in WP2 to explore the impact of each out-of-hospital service on unplanned hospital admissions. This will involve tracking a period before and after the commencement of each service and assessing any changes in the mean unplanned hospital admissions over time. To compare the costs and outcomes in patients who did (intervention) and did not (control) receive the out-of-hospital service, coarsened exact matching (CEM) and then a comparison of the differences in the mean costs and outcomes on the matched data will be performed. CEM temporarily coarsens the intervention patient demographic data (e.g. using coarse age groups rather than exact birthdays) and then finds control patients that are exact matches to the intervention patients. For each CEM application, standardised differences in the mean values will be estimated to assess balance in the variables used to match the intervention and control groups. Each intervention patient will be matched with up to 3 control patients. The 1:3 matching ratio will maximise the statistical power to detect a difference in outcomes between the intervention and control.

The QIC in WP3 will run from December 2020 to July 2021. Data analysis in WP3 will involve both quantitative and qualitative methods. Changes in adherence to selected process indicators within each service and pre-post levels of knowledge in collaborative members will be analysed using descriptive statistics. Qualitative interview and focus group data will be subject to inductive, thematic analysis [43].

## Discussion

Reducing rehospitalisations in older people is complex and requires sophisticated knowledge translation strategies [44, 45]. Accordingly, our protocol incorporates and interlinks epidemiology, health economics, and quality improvement methodologies. Further, we are engaging with a myriad of different stakeholder groups, which will enable an iterative approach and appropriate contextualisation throughout the program of work. Applying a novel and innovative approach, our aim is to address a complex problem at a state health system level in a way that recognises and accommodates differences in resources, knowledge, skills, and culture. Once established, the network will provide a platform of knowledge users, researchers, and policy makers that has the potential to tackle other complex problems associated with caring for the older population across SA.

## Data Availability

Both the quantitative and qualitative data will be stored on institutional network drives with security measures and approved servers in place. Hard copy records will be stored in a locked cabinet in a secure location. Access to records and data will be limited only to the research team. Study data will be de-identified and a master linking log with identifiers will be kept and stored separately from the data.

## Abbreviations

SA: South Australia
ROSA: Registry of Senior Australians
NGO: Non-government Organisation
iKT: Integrated Knowledge Translation
ED: Emergency Department
QIC: Quality Improvement Collaborative
LHN: Local Health Network
ACAT: Aged Care Assessment Team
SAHMRI: South Australian Health and Medical Research Institute
AIHW: Australian Institute of Health and Welfare
IQR: Interquartile Ranges
ITS: Interrupted Time Series
CEM: Coarsened Exact Matching
